# The Complete Chloroplast Genome Sequence of a Relict Conifer *Glyptostrobus pensilis*: Comparative Analysis and Insights into Dynamics of Chloroplast Genome Rearrangement in Cupressophytes and Pinaceae

**DOI:** 10.1371/journal.pone.0161809

**Published:** 2016-08-25

**Authors:** Zhaodong Hao, Tielong Cheng, Renhua Zheng, Haibin Xu, Yanwei Zhou, Meiping Li, Fengjuan Lu, Yini Dong, Xin Liu, Jinhui Chen, Jisen Shi

**Affiliations:** 1 Key Laboratory of Forest Genetics and Biotechnology, Ministry of Education, Nanjing Forestry University, Nanjing, China; 2 Co-Innovation Center for the Sustainable Forestry in Southern China, Nanjing, China; 3 College of Biology and the Envirionment, Nanjing Forestry University, Nanjing, China; 4 Southern Mountain Timber Forest Cultivation Lab, Fujian Academy of Forestry, Ministry of Forestry, Fuzhou, China; Austrian Federal Research Centre for Forests BFW, AUSTRIA

## Abstract

*Glyptostrobus pensilis*, belonging to the monotypic genus *Glyptostrobus* (Family: Cupressaceae), is an ancient conifer that is naturally distributed in low-lying wet areas. Here, we report the complete chloroplast (cp) genome sequence (132,239 bp) of *G*. *pensilis*. The *G*. *pensilis* cp genome is similar in gene content, organization and genome structure to the sequenced cp genomes from other cupressophytes, especially with respect to the loss of the inverted repeat region A (IRA). Through phylogenetic analysis, we demonstrated that the genus *Glyptostrobus* is closely related to the genus *Cryptomeria*, supporting previous findings based on physiological characteristics. Since IRs play an important role in stabilize cp genome and conifer cp genomes lost different IR regions after splitting in two clades (cupressophytes and Pinaceae), we performed cp genome rearrangement analysis and found more extensive cp genome rearrangements among the species of cupressophytes relative to Pinaceae. Additional repeat analysis indicated that cupressophytes cp genomes contained less potential functional repeats, especially in Cupressaceae, compared with Pinaceae. These results suggested that dynamics of cp genome rearrangement in conifers differed since the two clades, Pinaceae and cupressophytes, lost IR copies independently and developed different repeats to complement the residual IRs. In addition, we identified 170 perfect simple sequence repeats that will be useful in future research focusing on the evolution of genetic diversity and conservation of genetic variation for this endangered species in the wild.

## Introduction

*Glyptostrobus pensilis* (Staunton ex D. Don) K. Koch, also known as Chinese swamp cypress, is the only living species in the genus *Glyptostrobus*. *G*. *pensilis* is a typical tertiary relict species that was formerly very widespread in the northern hemisphere and then was reduced to its refugium before and during the Quaternary glaciations [1]. *G*. *pensilis* typically grows in deltas, near or in water, where it develops cypress knees acting as pneumatophores thought to help in oxygenation to the roots just like its related genus *Taxodium* [2, 3]. Disturbances from human activities (e.g., agriculture) over many years caused further reduction of its natural habitat [4]. The International Union for the Conservation of Nature (IUCN) Red List of Threatened Species has listed *G*. *pensilis* as Critically Endangered under criterion C in 2011, the highest threat category [5], and it is under first-grade state protection in China [6, 7]. At present, this species survives only in southeastern China, central Vietnam and possibly eastern Lao People’s Democratic Republic. Fortunately, *G*. *pensilis* has recently become the focus of increased attention, and more research on this endangered species is being carried out [8].

The chloroplast (cp) was once a free-living cyanobacterium that evolved into an intracellular organelle through at least two independent secondary endosymbiotic events [9]. Following endosymbiosis, the size of cp genomes was dramatically reduced as a result of many plastid-to-nucleus transfers [10, 11]. Since the first reports of the cp genome sequences from tobacco [12] and liverwort [13], cp genome sequences for an increasing number of plant species have been determined, especially with the development of next-generation sequencing in recent years. To date, more than 800 plant cp genome sequences have been deposited in the US National Center for Biotechnology Information (NCBI) Organelle Genome Resources (http://www.ncbi.nlm.nih.gov/genome/organelle/). Increasingly, these cp genome sequences are being used to obtain greater phylogenetic resolution, which is an effective approach to analyze plant phylogeny and population genetics [14–16].

Most plant cp genomes have a conserved quadripartite structure, with a pair of large inverted repeats (IRs) that divide the genome into large and small single-copy (LSC and SSC, respectively) regions [17, 18]. The large IRs, one of the distinguishing features in most cp genomes, range from 6 to 76 Kb in length [19] and play very important roles in stabilizing cp genome organization [20] and influencing cp genome size [21]. However, the large IRs have been lost from the cp genomes of species within tribes of the legume family (Fabaceae) and conifers, resulting in extensive rearrangements [20, 22]. Within conifers, two independent losses of an IR copy occurred in the cp genomes of Pinaceae and cupressophytes [23] since they separated from each other ~300 Mya [24, 25]. Here, we present the complete cp genome sequence (132,239 bp) of *G*. *pensilis*. We used the *G*. *pensilis* cp genome in conjunction with those of other conifer species to analyze rearrangements within cp genomes from Pinaceae and cupressophytes that occurred frequently after loss of the complete IRs, and we suggest possible explanations for differences in cp genome rearrangements between these two conifer lineages.

## Materials and Methods

### DNA extraction, sequencing and assembly

Fresh, young leaves were harvested from an adult plant of *G*. *pensilis* grown in Fuzhou National Forest Park with the permission of Fujian Provincial Department of Forestry (China). We washed and weighed out 20 g of leaves and then used the high-salt concentration method [26] to extract cp DNA. A 500-bp paired-end library was constructed using 5 μg of the isolated cp DNA. Approximately 2 GB of sequence, with an average read length of 301 bp, was obtained on the Illumina MiSeq platform.

To remove potential low-quality bases, raw reads were trimmed to 200 bp in length using an in-house ‘fasta_length_trimmer’ script. Then, clean reads were assembled *de novo* using Velvet Assembler version 1.2.07 [27]. Initial contigs were analyzed by performing a BLASTn search against NCBI nr/nt database. Contigs were collected for genome assembly if they showed high similarity to the published cp genome sequences, with E-value < 1e-10. We linked these contigs with paired-end MiSeq reads using SSPACE Premium version 2.2 [28] with a manual check. The structures of regions containing three pairs of longest repeats were validated by PCR amplicons with specific primers and Sanger sequencing on ABI 3730 DNA sequencers (S1 Table). Finally, one single circular cp genome sequence (132,239 bp) without ambiguous bases (N) was finally obtained.

### Genome annotation and sequence statistics

We used the online program Dual Organellar GenoMe Annotator (DOGMA) [29] for genome annotation followed by a manual check for exact boundaries of genes based on comparisons with their homologous genes in other sequenced conifer cp genomes. All transfer RNA (tRNA) genes were further confirmed using tRNAscan-SE version 1.21 [30] with default settings. We submitted the *G*. *pensilis* cp genome sequence to NCBI (accession number: KU302768) via Sequin version 13.70. The circular *G*. *pensilis* cp genome map was drawn using the OGDRAW program [31]. Codon usage and GC content at each of the three codon positions throughout the *G*. *pensilis* cp protein-coding genes were analyzed by MEGA6 [32].

### Construction of phylogenetic trees

For phylogenetic analysis, we downloaded 39 complete cp genomes of coniferous species representing three orders (Cupressales, Araucariales and Pinales) within Pinidae, as well as two other species, *Ginkgo biloba* and *Cycas revolute*, as outgroups (S2 Table). First, we extracted all genes in these cp genomes and reannotated any missing or abnormal gene annotations by comparison of conserved gene content and multiple sequence alignments (S3 Table). Next, we selected all 64 common protein-coding genes from these cp genomes and implemented a multiple sequence alignment of each set of orthologous genes using Clustal Omega version 1.2.0 with the “auto” option [33]. Then, each orthologous gene alignment was trimmed using trimAL version 1.2 with the “automated1” option, which is optimized for maximum likelihood (ML) phylogenetic tree reconstruction [34]. After that, we used an entropy-based index [35] implemented in DAMBE version 5.3.19 [36, 37] with the option of the proportion of invariant sites calculated by MEGA6 [32] to exclude orthologous gene alignments which had experienced severe substitution saturation (S4 Table). Finally, we obtained 47 orthologous genes and then concatenated these genes to form a gene nucleotide sequence matrix of 35,895 bp for constructing the phylogenetic tree.

We performed phylogenetic analysis by ML based on the sequence matrix, using phyML version 3.1 [38]. We selected the custom option to implement a General Time Reversible + Proportion Invariant + Gamma (GTR + I + G) nucleotide substitution model that was selected as the best-fit model with–lnL of 282,045.8750 by Modeltest version 3.7 coupled with PAUP4b10 [39]. In addition, subtree pruning and regrafting (SPR) were performed to estimate tree topologies, with five random starting trees used for each standard BioNJ starting tree. The degree to which each internal branch of the phylogeny was supported by the data was estimated by 1000-replicate non-parametric bootstrap analysis.

### Genome rearrangement analysis

**Fig 1:**
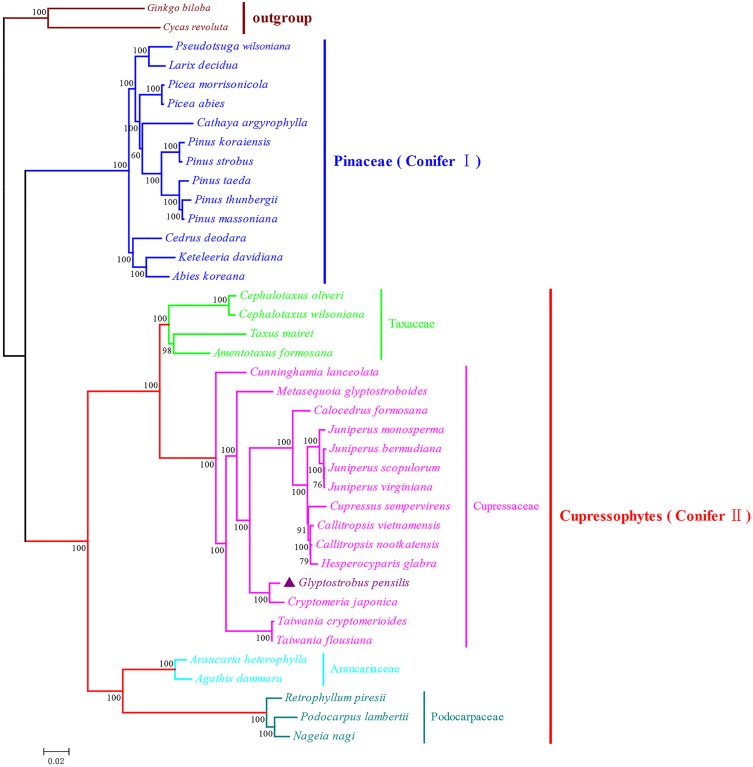
Phylogenetic analysis of conifer cp genomes. ML analysis was performed based on 47 cp protein-coding genes with a GTR + I + G model. *G*. *biloba* and *C*. *revolute* were set as outgroups. Support values for each branch based on a bootstrap analysis of 1,000 nonparametric replicates are shown. The scale of branch length is indicated in the bottom left corner.

The complete cp genome sequences of 36 coniferous species, 23 cupressophytes and 13 Pinaceae, and one rooted species, *G*. *biloba*, were downloaded from NCBI for comparison (S2 Table). As cp genome molecules are circular, we linearized these cp genomes so that the *psbA* gene was always at the start for easy comparison. The IRA and IRB regions of *G*. *biloba* cp genome were separately removed when compared to the clades cupressophytes and Pinaceae, respectively. Using progressive Mauve implemented in MAUVE version 2.4.0 [40], two matrices of cupressophytes and Pinaceae containing 26 and 7 locally collinear blocks (LCBs), respectively, were generated (S1 Data). The topologies of cupressophytes and Pinaceae inferred from the phylogenetic analysis (Fig 1) were used as suggested actual trees when we used MGR version 2.0.3 [41] to estimate the rearrangement events with the option of unichromosomal circular reversal distance based on the two matrices of LCBs. Finally, the number of rearrangement steps required for transforming cp genome of each species into that of *G*. *biloba* was calculated by adding all estimated numbers of rearrangements above the branches linking the corresponding species to *G*. *biloba*.

### Divergence time estimation

Based on the 47 protein-coding genes, we used MCMCTree in PAML to perform Bayesian estimation of species divergence times using soft fossil constraints under various molecular clock models [42]. The topology was constrained to reflect the ML tree, and a GTR substitution model was used. We incorporated seven fossil constraints, i.e., Conifer divergence, *Araucariaceae*—*Podocarpaceae* divergence, *Podocarpus*—*Retrophyllum* divergence, *Taxaceae*—*Cupressaceae* divergence, *Juniperus*—*Cupressus* divergence, *Picea—Cathaya* divergence, and *Larix*—*Pseudotsuga* divergence, and these constraints was set following Leslie *et al* [24]. The Markov chain Monte Carlo (MCMC) process of PAML mcmctree was run to sample 1, 000, 000 times, with sample frequency set to 50, after a burn-in of 500, 000 iterations.

### Repeat analysis

We identified palindromic repeats in 37 conifer cp genomes using the online program REPuter [43] with a cutoff value of 30 bp for each repeat unit and 3 for the Hamming distance (i.e., >90% identity) between a pair of repeat units. The Perl script MISA (http://pgrc.ipk-gatersleben.de/misa/) was used to identify simple sequence repeats (SSRs) in the *G*. *pensilis* cp genome with a minimum repeat count of eight for mononucleotide repeats, four for di- and trinucleotide repeats and three for tetra-, penta- and hexanucleotide repeats. All preliminary results from the various programs were manually checked to avoid redundancy, in that any two repeats we identified were not overlapped.

## Results and Discussion

### Chloroplast genome features of *G*. *pensilis*

The complete cp genome of *G*. *pensilis* is 132,239 bp in length, with an overall GC content of 35.31% (Table 1). The size of the *G*. *pensilis* cp genome is similar to those (127–146 Kb) of other sequenced cupressophytes [44]. As shown in S1 Fig, the *G*. *pensilis* cp genome is circular and lacks the typical quadripartite structure consisting of a pair of IRs separated by LSC and SSC regions. The structure of the complete IRs, which were lost from the cp genomes of other coniferous species [22, 23, 45], were also not found in the *G*. *pensilis* cp genome, therefore the LSC and SSC regions could not be defined in this cp genome. The *G*. *pensilis* cp genome encodes 119 genes, including 83 protein-coding genes, 32 tRNA genes and four ribosomal RNA (rRNA) genes (S5 Table). Among the 119 genes, 115 are single-copy genes, and two, *trnI-CAU* and *trnQ-UUG*, are duplicated (S5 Table). Of the 115 single-copy genes, 15 contain one intron (nine protein-coding genes and six tRNA genes) and two, *rps12* and *ycf3*, contain two introns (S5 and S6 Tables). In addition, *rps12* was identified as a trans-spliced gene, with the N-terminal exon I being located 92 Kb from the C-terminal exons II and III [46], and *trnK-UUU* has the longest intron (2,424 bp), which includes the *matK* gene (S6 Table).

**Table 1:** Chloroplast genome features of *G*. *pensilis*.

	T/U (%)	C (%)	A (%)	G (%)	Length (bp)	GC (%)
**Genome**	32.25	17.13	32.44	18.18	132,239	35.31
**tRNA genes**	24.10	24.63	22.84	28.43	2,452	53.06
**rRNA genes**	20.08	22.52	26.85	30.55	4,592	53.07
**Protein-coding genes**	31.61	16.87	31.64	19.87	73,959	36.74
**1st codon position**	23.35	18.29	30.71	27.65	24,653	45.94
**2nd codon position**	32.79	19.89	30.39	16.93	24,653	36.82
**3rd codon position**	38.70	12.45	33.80	15.05	24,653	27.50

Protein-coding regions, which contain 83 protein-coding genes, are 73,959 bp in length and account for 55.93% of the *G*. *pensilis* cp genome. Genes for rRNAs and tRNAs constitute 3.47% and 1.85% of the *G*. *pensilis* cp genome, respectively, and the remaining 38.75% of non-coding regions are comprised of intergenic spacers and introns. The GC content at the first, second and third codon positions of protein-coding genes is 45.94, 36.82 and 27.50%, respectively (Table 1). This trend of decreasing GC content at the three codon positions and the bias toward a lower GC content at the third codon position has been observed in many other sequenced plant cp genomes, and this pattern contributes to the relatively high AT content throughout the cp genome [47–51]. With regard to amino acid and codon usage, the most- and least-frequently coded amino acids are leucine (2660, 10.83%) and cysteine (279, 1.14%), respectively, whearea AAA (1176, 4.79%) and CGG (77, 0.31%) are the most and least used, respectively (S2 Fig and S7 Table).

### Phylogenetic analysis

*G*. *pensili*s, the only living species of the genus *Glyptostrobus*, is an aquatic endangered conifer that was widely distributed throughout the Northern Hemisphere in the Late Cretaceous and the Early Tertiary [52]. To test the phylogenetic position and evolutionary history of *G*. *pensili*s among conifers, we used ML based on a nucleotide sequence matrix of concatenated protein-coding genes to construct a phylogenetic tree showing the evolutionary relationships among coniferous species representing three orders within subclass Pinidae.

As shown in Fig 1, the constructed ML tree indicated two major conifer clades, conifer I and conifer II, with very high overall bootstrap values and in agreement with the results of previous studies [53, 54]. All 13 Pinaceae species are clustered in the conifer I clade, and the remaining coniferous species are clustered in the conifer II, namely cupressophytes. Within the cupressophyte clade, there are three major subclades (the Taxaceae subclade, the Cupressaceae subclade and the subclade comprised of Araucariaceae and Podocarpaceae), similar to the topology inferred from nuclear plastid DNA and their plastomic counterparts [55]. In the Cupressaceae subclade, *G*. *pensili*s and *Cryptomeria japonica* form a sister branch with 100% bootstrap support that is consistent with previous studies inferred from several cp genes [56, 57]. The placement of *G*. *pensili*s is in accord with the deduction inferred from fossil records and paleoclimatic data that the genus *Glyptostrobus* and *Taxodium* might originate from a common ancestor that had a close relationship with the genus *Cryptomeria* [1].

### Extensive rearrangements within cupressophyte cp genomes

In terms of gene content and organization, cp genomes are largely conserved relative to nuclear and mitochondrial genomes [58]. In angiosperms, the structure of cp genome is highly conserved, i.e., there is a typical quadripartite structure consisting of a LSC region and a SSC region separated by a pair of IRs [59]. In contrast, numerous genome rearrangements have been observed in several genera from the cupressophyte lineage, including *Cryptomeria* [60], *Agathis*, *Nageia* and *Calocedrus* [44]. Because coniferous species are classified into two groups in the phylogenetic tree (cupressophytes and Pinaceae; Fig 1), and conifers underwent two different processes of cp genome evolution after splitting ~300 Mya (S3 Fig) [24, 25], it is interesting to do research on the comparison of the cp genome rearrangements between these two major conifer clades.

**Fig 2:**
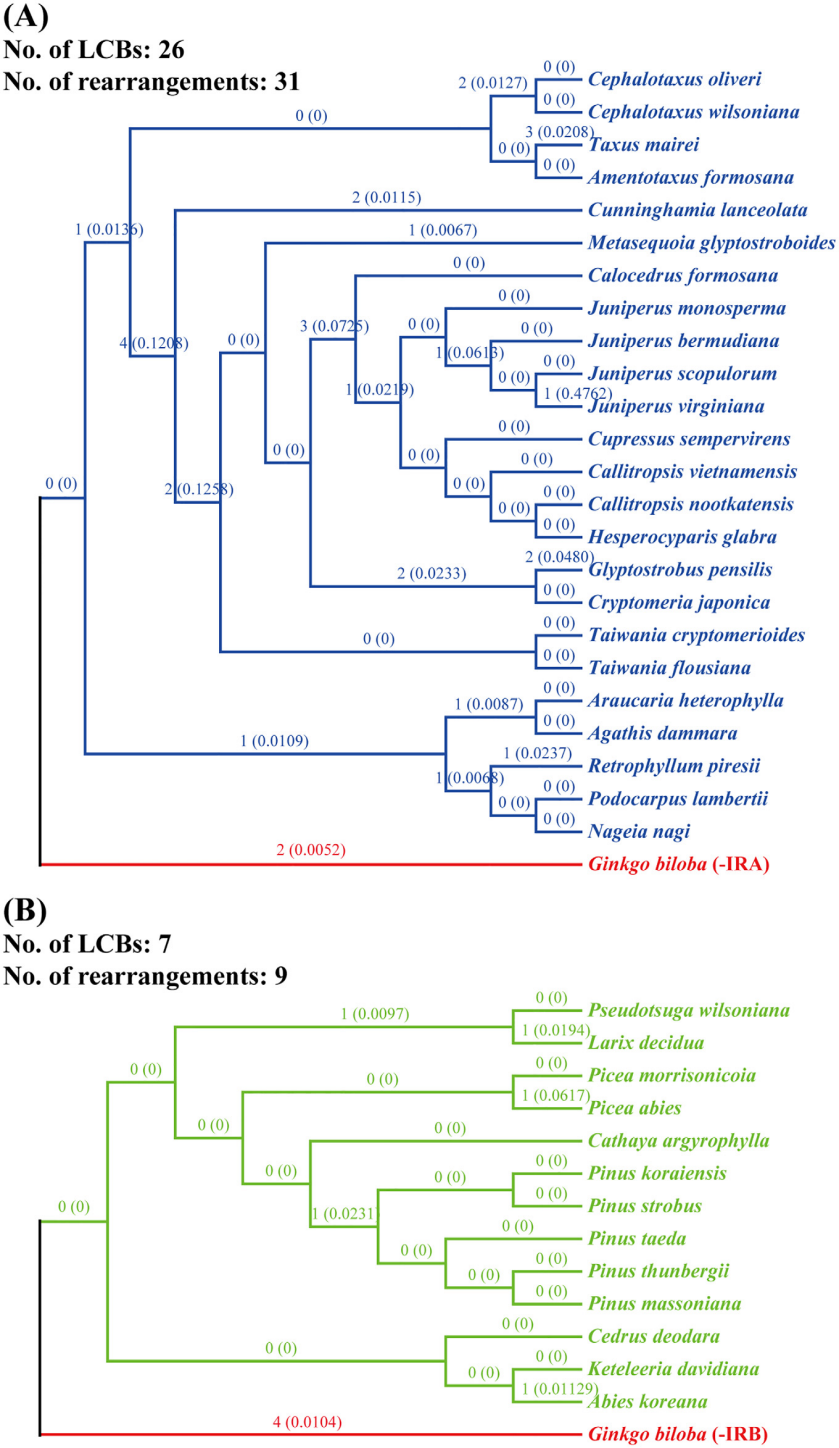
Chloroplast genome rearrangement estimates among cupressophytes and Pinaceae. The topologies of clades cupressophytes (A) and Pinaceae (B) constructed from phylogenetic analysis were used as suggested actual trees and rearrangements were inferred from the matrices of cp genome LCBs. The estimated number of rearrangements for branches to taxa are shown above branches and corresponding rearrangements per million years were shown in brackets. The cp genome of *G*. *biloba* with removing IRA and IRB separately was used as a rooted genome in these two comparisons, respectively.

The complete cp genomes of the two conifer groups, which contain 24 and 13 species, were separately compared. Considering that cupressophyte lost IRA whereas Pinaceae lost IRB, we used the complete cp genome of *G*. *biloba* as a root with removing IRA and IRB manually, respectively, in these two comparisons. Finally, two trees compatible with the topology inferred from phylogenetic analysis (Fig 1) were generated. As shown in Fig 2A, only two rearrangements were required to transform the cp genome of *G*. *pensilis* into that of *C*. *japonica*, suggesting a close relationship between these two species. In total, the number of rearrangements for the clade cupressophyte is 31, whereas the clade Pinaceae only required 9 rearrangements (Fig 2). Moreover, cupressophyte cp genomes diverged at a rate of approximately 0.1031 rearrangements per million years, whereas Pinaceae cp genomes diverged at a rate of approximately 0.0286 rearrangements per million years (Fig 2), which is indicative of extensive rearrangements in cp genomes of cupressophytes compared with Pinaceae. Because both cupressophyte and Pinaceae cp genomes have lost the complete IRs [23] and the Pinaceae-specific repeats were able to complement the residual IRs [61], we speculated that cupressophyte may have less functional repeats, leading to relatively extensive cp genome rearrangements that occurred in the evolutionary history of cupressophyte cp genomes.

### The *G*. *pensilis* cp genome lost inverted repeat region A (IRA)

The *G*. *pensilis* cp genome lacks the typical quadripartite structure (S1 Fig) because of the loss of one IR copy, which has also been observed in other conifer cp genomes from Pinaceae [22, 45] and cupressophytes [51, 60]. Since cp genomes of these two conifer clades lost different IR copies [23, 44], comparsion of cp genome structure were performed to confirm which one IR copy were lost in the *G*. *pensilis* cp genome.

**Fig 3:**
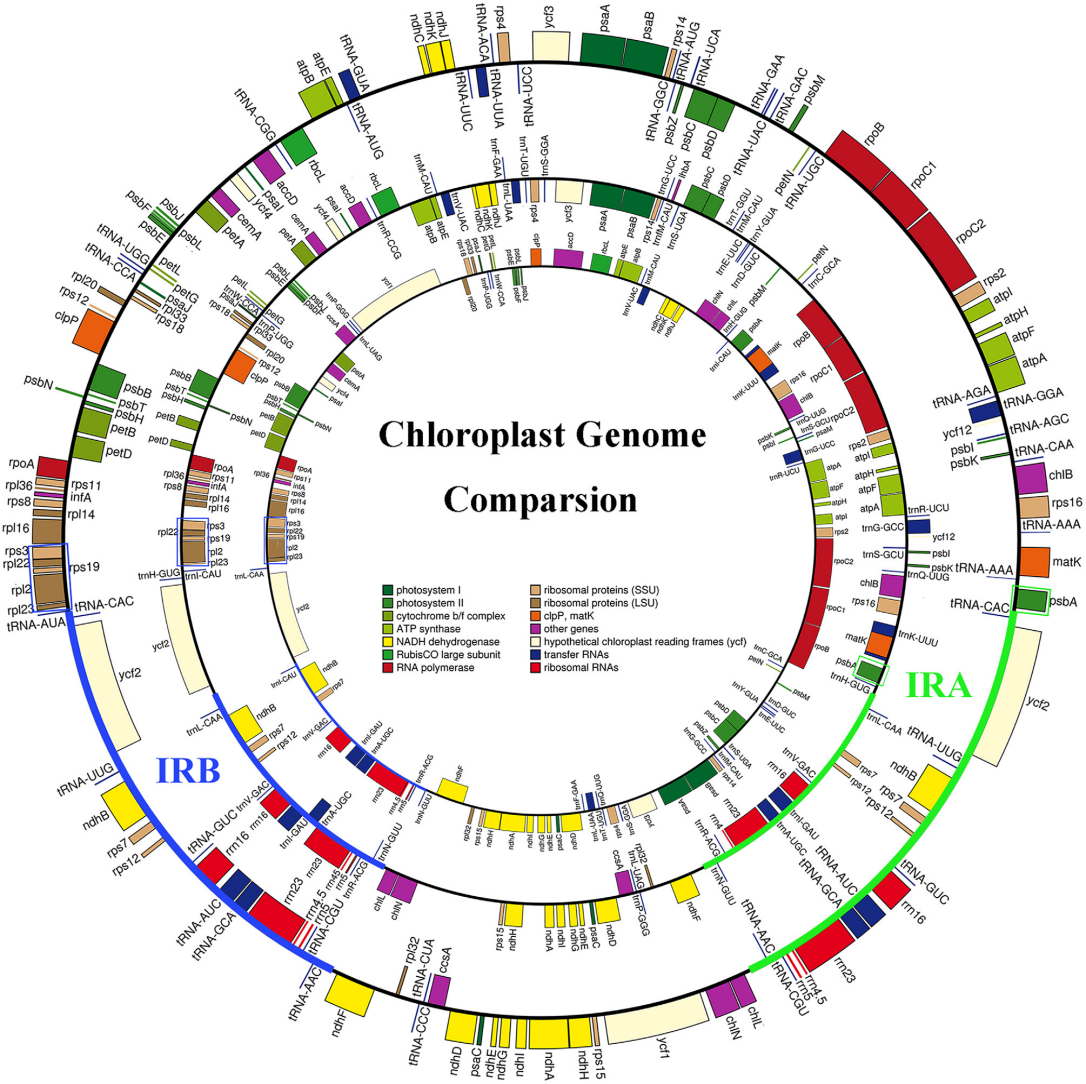
The *G*. *pensilis* cp genome lost IRA in comparison with *G*. *biloba* and *C*. *revolute*. Outer to inner circles correspond to the cp genome maps of *C*. *revolute*, *G*. *biloba* and *G*. *pensilis*, respectively. The bold blue and green lines in the *C*. *revolute* and *G*. *biloba* cp genome circles correspond to the IRA and IRB regions, respectively. The blue solid boxes correspond to the *rpl23*-*rps3* cluster, which is always downstream of the IRB region, and the green solid boxes correspond to *psbA*, which is always upstream of the IRA region in a clockwise direction.

As shown in Fig 3, the IR region of the *C*. *revolute* and *G*. *biloba* cp genomes always contains the rRNA operon, six tRNA genes (*trnN-GUU*, *trnR-ACG*, *trnA-UGC*, *trnI-GAU*, *trnV-GAC* and *trnL-CAA*) and three protein-coding genes (*ndhB*, *rps7* and *rps12*). The IR region of *G*. *biloba* has shrunken compared with that of *C*. *revolute*, losing *ycf2* and *trnH-GUG*. However, gene content and gene order are highly conserved near the junctions of the LSC region with IRA and inverted repeat region B (IRB) that, moving clockwise, *psbA* (green solid boxes in Fig 3) is always upstream of the IRA and the *rpl23*-*rps3* cluster (blue solid boxes in Fig 3) is always downstream of the IRB. This type of conserved gene order has been informative for the identification of IR copies lost from cp genomes of coniferous species [23]. In the *G*. *pensilis* cp genome, we found that the rRNA operon is not duplicated and the gene segment containing the rRNA operon (blue line in the *G*. *pensilis* cp genome map in Fig 3) is adjacent to the *rpl23*-*rps3* cluster. The data presented here strongly suggest that, in the *G*. *pensilis* cp genome, the lost IR copy is very likely to be the IRA, rather than the IRB.

### Less functional repeats within cupressophyte cp genomes

**Fig 4:**
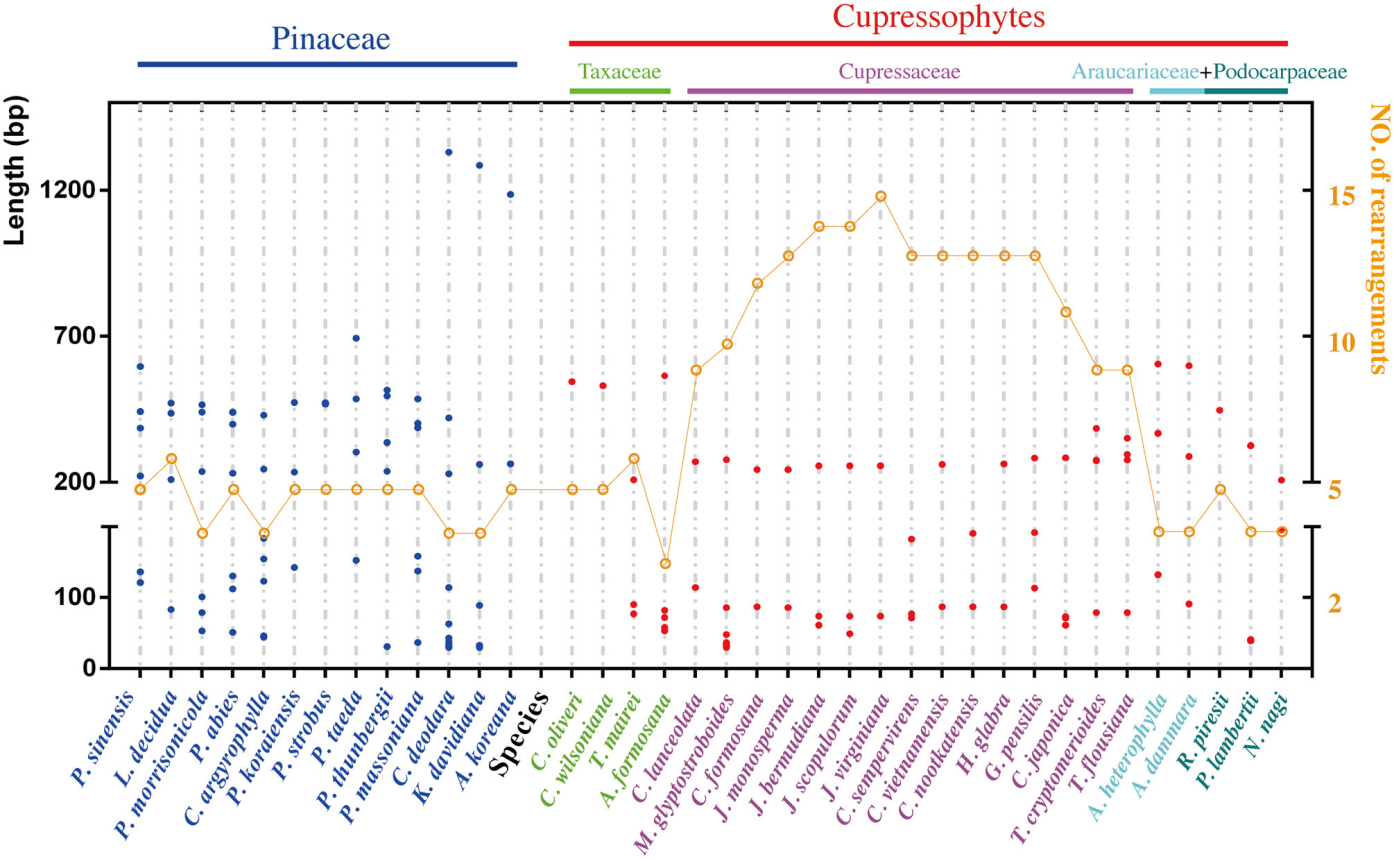
The distribution of palindromic repeats and estimated number of rearrangement events in conifer cp genomes. The full binomial species names for the species in this figure are listed in S2 Table. Dots colored in blue and red belong to the Pinaceae and cupressophytes, respectively and represent a pair of palindromic repeats with the size of the repeat unit corresponding to the value on the left y-axis. Dots colored in orange represent the estimated number of rearrangement events required for transforming corresponding species cp genome into that of *G*.*biloba* and correspond to the value on the right y-axis.

The large IRs play an important role in maintaining a conserved arrangement and stabilizing the cp genome [20]. During the evolution of the angiosperms, one IR copy was lost in the cp genomes of tribes in the legume subfamily Papilionoideae [62–64], and cp DNA rearrangements are more frequent in these species relative to those with the normal IRs [20]. In coniferous species, the complete IRs was lost in both Pinaceae and cupressophyte cp genomes, and the conifer cp genomes have many more rearrangements as compared with most higher plants [22]. The residual IR in the cp genome was proved to be different between Pinaceae and cupressophyte, suggesting that these two conifer clades lost one IR copy independently in their own evolutionary history after they split from a common ancestor [23, 44]. Since cp genome rearrangements were more extensive in cupressophyte than in Pinaceae (Fig 2) and Pinaceae-specific repeats could replace the reduced IRs [61], it is interesting to deep explore the influences of potential functional repeats in conifer cp genome rearrangement dynamics. We identified palindromic repeats within 37 conifer cp genomes, 13 from Pinaceae and 24 from cupressophytes (S8 Table). Fig 4 depicts the distribution of palindromic repeats in these 37 cp genomes of coniferous species. The palindromic repeats in the *G*. *pensilis* cp genome have similar characteristics to those of other cupressophyte species. Overall, repeats that were <200 bp had a similar distribution in the Pinaceae and cupressophyte cp genomes, ranging from zero to four or five across species. In contrast, there was a distinct difference between Pinaceae and cupressophyte species in terms of palindromic repeats with length greater than 200 bp, in that there are more of these repeats and they are longer in the former than in the latter. Within the cupressophyte calde, species of the subclade Cupressaceae all have a high number of rearrangements with relatively shorter repeats compared to the species in other two subclades. Previous cp genome transformation studies have shown that repeats >200 bp are effective substrates for homologous recombination [65], and evolution endowed a novel type of repeat that could replace the highly reduced IRs in Pinaceae cp genomes [61]. As there are more potential functional repeats in cp genomes of Pinaceae than in those of cupressophytes, this might explain, at least in part, the phenomenon that cp genome rearrangements are much more frequent in cupressophytes, espically in the subclade Cupressaceae, than in Pinaceae.

**Fig 5:**
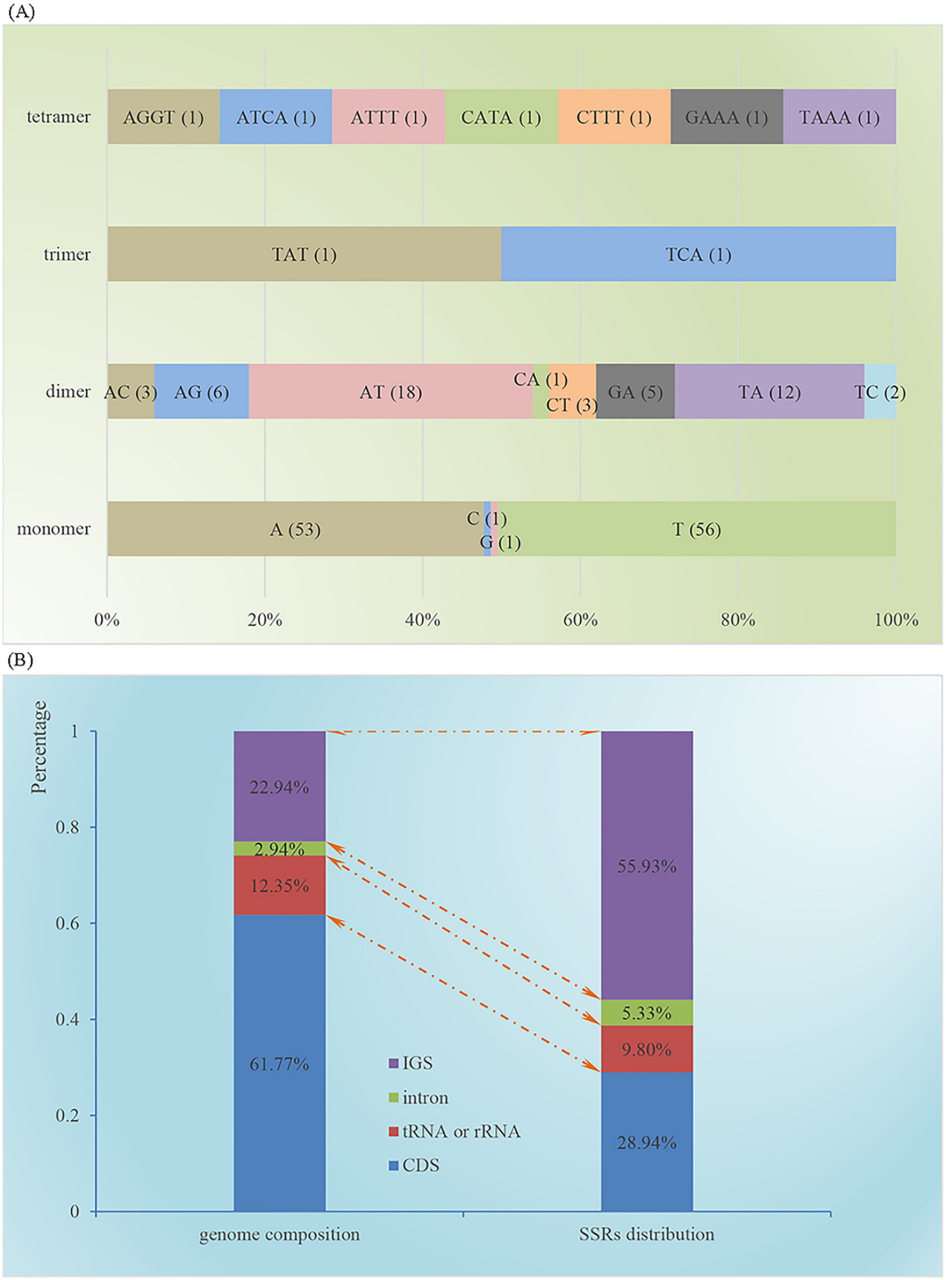
SSR analysis in the *G*. *pensilis* cp genome. (A) Frequency of identified SSR motifs in different repeat type classes. There were four kinds of SSR motifs identified in the *G*. *pensilis* cp genome. Most of the mononucleotides (109 of 111) are composed of A/T, and the majority of dinucleotides (30 of 50) are composed of AT/TA, whereas the other two kinds of SSRs have a high A/T content. (B) *G*. *pensilis* cp genome composition and SSR motifs distribution. SSRs are more abundant in the intergenic sequences (IGS, 55.93%) than in protein-coding regions (CDS, 28.94%), which account for 22.94 and 61.77% of the *G*. *pensilis* cp genome, respectively.

In addition, we identified 170 perfect SSRs in the *G*. *pensilis* cp genome: 111 mononucleotide, 50 dinucleotide, two trinucleotide, and seven tetranucleotide repeats (S9 Table). As shown in Fig 5, SSRs had high A/T content and were unevenly distributed in the *G*. *pensilis* cp genome. Although different algorithms and criteria were used for SSR identification, their characteristics and distribution were similar to those reported for other conifer cp genomes [51, 66], 14 monocot cp genomes [67] and 30 asterid cp genomes [50]. The SSRs we have identified in the *G*. *pensilis* cp genome can be assessed for the polymorphism at the intraspecific level for being used as molecular markers to study the genetic diversity and genetic structure of natural populations of this endangered species.

## Conclusion

We used Illumina sequencing followed by *de novo* assembly to obtain the complete cp genome sequence (132,239 bp) of the endangered species *G*. *pensilis*. *Glyptostrobus* is a monotypic genus that, based on analysis of some physiological characteristics, appears to be closely related to the genera *Taxodium* and *Cryptomeria* [1]. Our phylogenetic tree, constructed using concatenation of multiple cp protein-coding genes, provides further support for this relationship. The cp genome map of *G*. *pensilis* indicates that there are no large IRs and further cp genome comparison suggests that one IR copy, most likely the IRA, was lost from the *G*. *pensilis* cp genome. In addition, there were many more rearrangements in cp genomes of cupressophyte species than in those of Pinaceae species, which could be related to the distribution of palindromic repeats (>200 bp) in these two major conifer clades. After IRB were lost from the cp genomes of Pinaceae, evolution endowed this conifer clade specific repeats to complement the residual IRs [61]. Although similar repeats were also found in the cupressophyte cp genomes [47], our results indicated that this conifer clade contained less potential functional repeats after losing IRA, leading to relatively extensive cp genome rearrangements compared to Pinaceae. We anticipate that the results presented here will be helpful both for deeper research on this endangered species and greater understanding of the complex evolutionary history of conifer cp genomes.

## Supporting Information

S1 Data Matrices of LCBs (locally co-linear blocks) for computing multiple cp genome rearrangement scenarios. (PDF)

S1 Fig The cp genome map of the *G*. *pensilis*. Genes are transcribed clockwise (inside of the circle) and counter-clockwise (outside of the circle), respectively. Genes classified to different functional groups are color-coded corresponding to the table on the bottom left corner. The next circle denotes the GC content represented on the inner circle by dark gray bars and AT content represented on the outer circle by lighter gray bars, respectively.
(TIF)

S2 Fig The distribution of amino acids and codons within the *G*. *pensilis* cp protein-coding genes. The number of each amino acid and corresponding codons were calculated for all of the 83 protein-coding genes from the start codon to the stop codon in the *G*. *pensilis* cp genome excluding introns and stop codons. Leucine dotted purple box and cysteine dotted green box were the most and least coded amino acids, respectively. AAA dotted red box and CGG dotted blue box were the most and the least used codons, respectively.
(TIF)

S3 Fig Dated phylogeny for 37 conifer species with ginkgo and cycads as outgroups. A time scale is shown at the bottom and these colored rectangles indicate different geological periods. The red points in some nodes indicate fossil calibration points.
(TIF)

S1 Table The validation results for the structures of regions containing three pairs of longest repeats by PCR amplicons and Sanger sequencing. (DOCX)

S2 Table GenBank accession numbers of the cp genomes used in this study. (DOCX)

S3 Table Reannotation of missing (written in red) or mistaken (written in blue) annotations by comparison of conserved gene content and order. (XLSX)

S4 Table The index of substitution saturation (Iss) values of 64 protein-coding genes common to 39 species. (DOCX)

S5 Table Genes present in the *Glyptostrobus pensilis* chloroplast genome. (DOCX)

S6 Table Genes with introns in the *G*. *pensilis* cp genome. (DOCX)

S7 Table The codon-anticodon recognition pattern and codon usage for the *G*. *pensilis* cp genome. (DOCX)

S8 Table Palindromic repeats identified in 37 conifer cp genomes by using REPuter with the cutoff value of 30 bp. (XLSX)

S9 Table Distribution of SSRs present in *G*. *pensilis* chloroplast genome. (XLSX)
