# Supplementary material for: The Complete Chloroplast Genome Sequence of a Relict Conifer Glyptostrobus pensilis: Comparative Analysis and Insights into Dynamics of Chloroplast Genome Rearrangement in Cupressophytes and Pinaceae
Source: PLoS One. 2016 Aug 25;11(8):e0161809. doi: 10.1371/journal.pone.0161809 (PMC4999192; doi:10.1371/journal.pone.0161809)
Supplement: S1 Data — (PDF) [file pone.0161809.s001.pdf]

S1 Data. Matrices of LCBs (locally co-linear blocks) for computing multiple cp genome rearrangement scenarios.

-- cupressophytes --

>Cryptomeria japonica

1,2,3,7,6,5,4,8,24,26,21,20,19,23,25,13,17,18,16,15,14,12,11,10,9,22$

>Glyptostrobus pensilis

1,2,3,-4,-5,-6,-7,8,24,26,21,20,19,-18,-17,-13,-25,-23,16,15,14,12,11,10,9,22$

>Cunninghamia lanceolata

1,2,3,7,6,5,4,20,19,-18,-17,-13,-14,-15,-16,23,25,24,26,21,8,12,11,10,9,22$

>Taiwania flousiana

1,2,3,7,6,5,4,8,24,26,21,20,19,-18,-17,-13,-14,-15,-16,23,25,12,11,10,9,22$

>Taiwania cryptomerioides

1,2,3,7,6,5,4,8,24,26,21,20,19,-18,-17,-13,-14,-15,-16,23,25,12,11,10,9,22$

>Metasequoia glyptostroboides

1,2,3,-4,-5,-6,-7,8,24,26,21,20,19,-18,-17,-13,-14,-15,-16,23,25,12,11,10,9,22$

>Taxus mairei

1,2,3,7,-6,5,4,8,-9,-10,-11,-12,-17,-13,-14,-15,-16,18,19,-20,-21,-26,25,24,-23,22$

>Araucaria heterophylla

1,2,3,-4,-5,-6,-7,8,-9,-10,-11,-12,-13,-14,15,-16,17,18,23,-24,-25,26,21,20,-19,22$

>Agathis dammara

1,2,3,-4,-5,-6,-7,8,-9,-10,-11,-12,-13,-14,15,-16,17,18,23,-24,-25,26,21,20,-19,22$

>Retrophyllum piresii

1,2,3,-4,-21,-26,25,24,-23,17,18,16,15,14,13,12,11,10,9,-8,7,6,5,20,-19,22$

>Podocarpus lambertii

1,2,3,-4,-5,-6,-7,8,-9,-10,-11,-12,-13,-14,-15,-16,-18,-17,23,-24,-25,26,21,20,-19,22$

>Nageia nagi

1,2,3,-4,-5,-6,-7,8,-9,-10,-11,-12,-13,-14,-15,-16,-18,-17,23,-24,-25,26,21,20,-19,22$

>Amentotaxus formosana

1,2,3,-4,-5,-6,-7,8,-9,-10,-11,-12,16,15,14,13,17,18,19,-20,-21,-26,25,24,-23,22$

>Cephalotaxus wilsoniana

1,-22,23,-24,-25,26,21,20,-19,-18,-17,-13,-14,-15,-16,12,3,-4,-5,-6,-7,8,-9,-10,-11,-2$

>Cephalotaxus oliveri

1,-22,23,-24,-25,26,21,20,-19,-18,-17,-13,-14,-15,-16,12,3,-4,-5,-6,-7,8,-9,-10,-11,-2$

>Juniperus scopulorum

1,2,3,7,6,5,4,8,24,26,21,20,19,23,-25,16,15,14,13,17,18,12,11,10,9,22$

>Juniperus bermudiana

1,2,3,7,6,5,4,8,24,26,21,20,19,23,-25,16,15,14,13,17,18,12,11,10,9,22$

>Calocedrus formosana

1,2,3,7,6,5,4,8,24,26,21,20,19,23,-25,16,15,14,13,17,18,12,11,10,9,22$

>Juniperus virginiana

1,2,3,-4,-5,-6,-7,8,24,26,21,20,19,23,-25,16,15,14,13,17,18,12,11,10,9,22$

>Juniperus monosperma

1,2,3,-4,-5,-6,-7,8,24,26,21,20,19,23,-25,16,15,14,13,17,18,12,11,10,9,22$

>Hesperocyparis glabra

1,2,3,-4,-5,-6,-7,8,24,26,21,20,19,23,-25,16,15,14,13,17,18,12,11,10,9,22$

>Cupressus sempervirens

1,2,3,-4,-5,-6,-7,8,24,26,21,20,19,23,-25,16,15,14,13,17,18,12,11,10,9,22$

>Callitropsis vietnamensis

1,2,3,-4,-5,-6,-7,8,24,26,21,20,19,23,-25,16,15,14,13,17,18,12,11,10,9,22$

>Callitropsis nootkatensis

1,2,3,-4,-5,-6,-7,8,24,26,21,20,19,23,-25,16,15,14,13,17,18,12,11,10,9,22$

>Ginkgo biloba (-IRA)

1,2,3,-4,-5,-6,-7,8,-9,10,-11,-12,-13,-14,-15,-16,17,18,19,-20,-21,-22,23,-24,-25,26$

-- Pinaceae --

>Larix decidua

1,4,2,5,3,7,6$

>Abies koreana

1,-4,2,5,3,7,6$

>Pice abies

1,-4,2,5,3,7,6$

>Pseudotsuga sinensis

1,-4,2,5,3,7,6$

>Cathaya argyrophylla

1,-2,4,5,3,7,6$

>Cedrus deodara

1,-2,4,5,3,7,6$

>Keteleeria davidiana

1,-2,4,5,3,7,6$

>Picea morrisonicoia

1,-2,4,5,3,7,6$

>Pinus koraiensis

1,-2,-4,5,3,7,6$

>Pinus massoniana

1,-2,-4,5,3,7,6$

>Pinus strobus

1,-2,-4,5,3,7,6$

>Pinus taeda

1,-2,-4,5,3,7,6$

>Pinus thunbergii

1,-2,-4,5,3,7,6$

>Ginkgo biloba (-IRB)

1,-2,-3,4,5,6,-7$
