# Supplementary figures and images for: The Complete Chloroplast Genome Sequence of a Relict Conifer Glyptostrobus pensilis: Comparative Analysis and Insights into Dynamics of Chloroplast Genome Rearrangement in Cupressophytes and Pinaceae
Source: PLoS One. 2016 Aug 25;11(8):e0161809. doi: 10.1371/journal.pone.0161809 (PMC4999192; doi:10.1371/journal.pone.0161809)

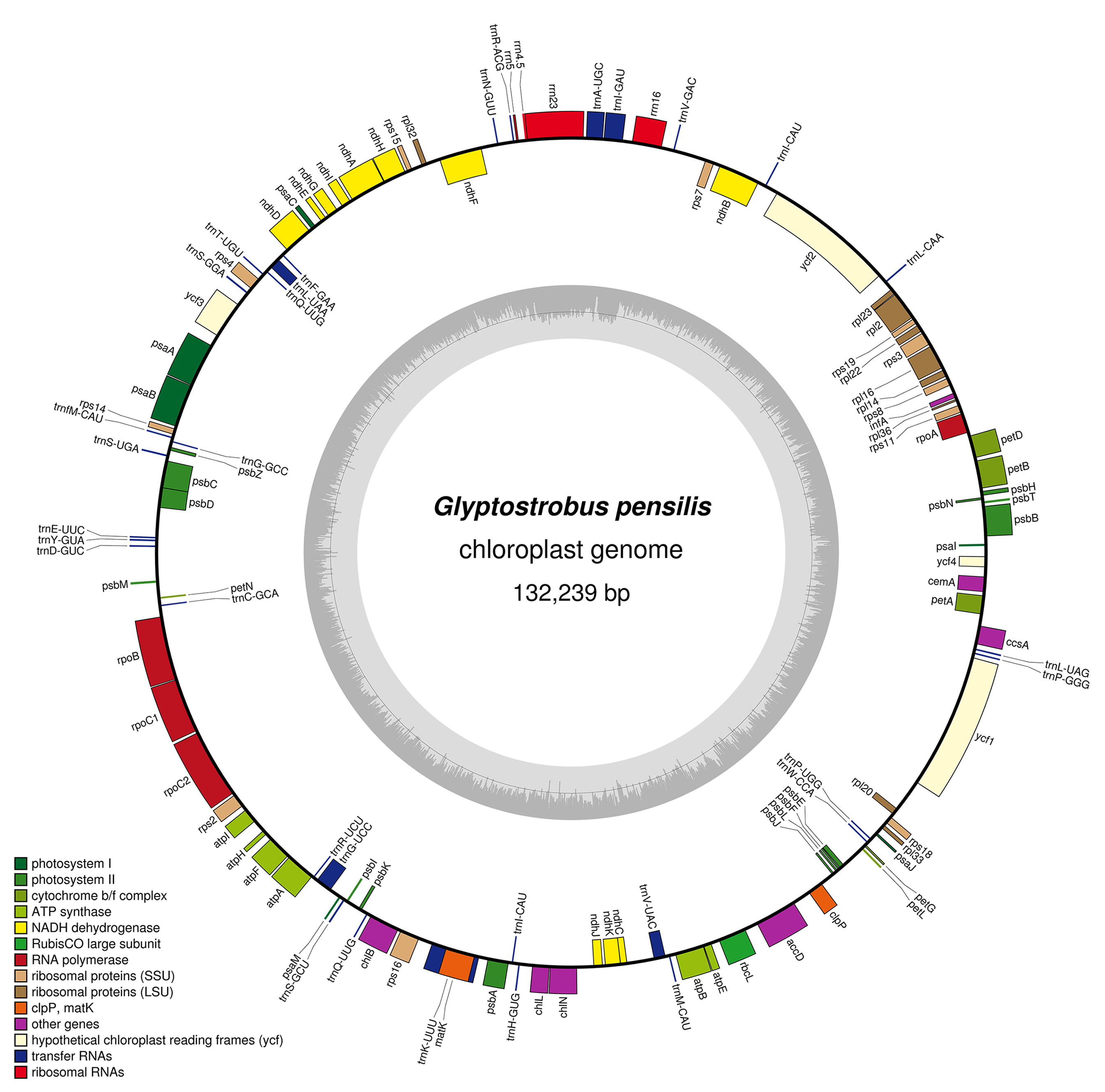

Supplement: S1 Fig — Genes are transcribed clockwise (inside of the circle) and counter-clockwise (outside of the circle), respectively. Genes classified to different functional groups are color-coded corresponding to the table on the bottom left corner. The next circle denotes the GC content represented on the inner circle by dark gray bars and AT content represented on the outer circle by lighter gray bars, respectively. (TIF) [file pone.0161809.s002.tif]

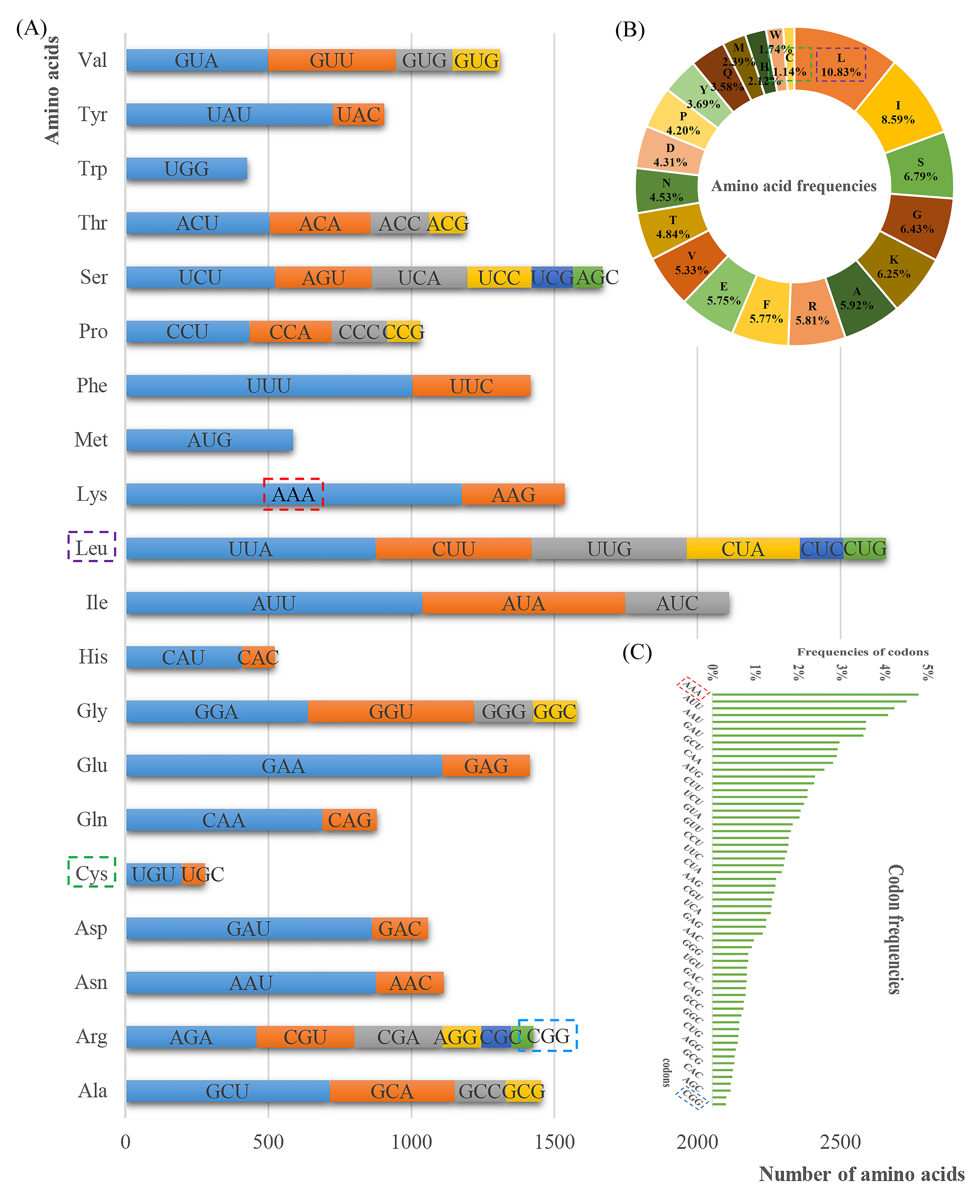

Supplement: S2 Fig — The number of each amino acid and corresponding codons were calculated for all of the 83 protein-coding genes from the start codon to the stop codon in the G. pensilis cp genome excluding introns and stop codons. Leucine dotted purple box and cysteine dotted green box were the most and least coded amino acids, respectively. AAA dotted red box and CGG dotted blue box were the most and the least used codons, respectively. (TIF) [file pone.0161809.s003.tif]

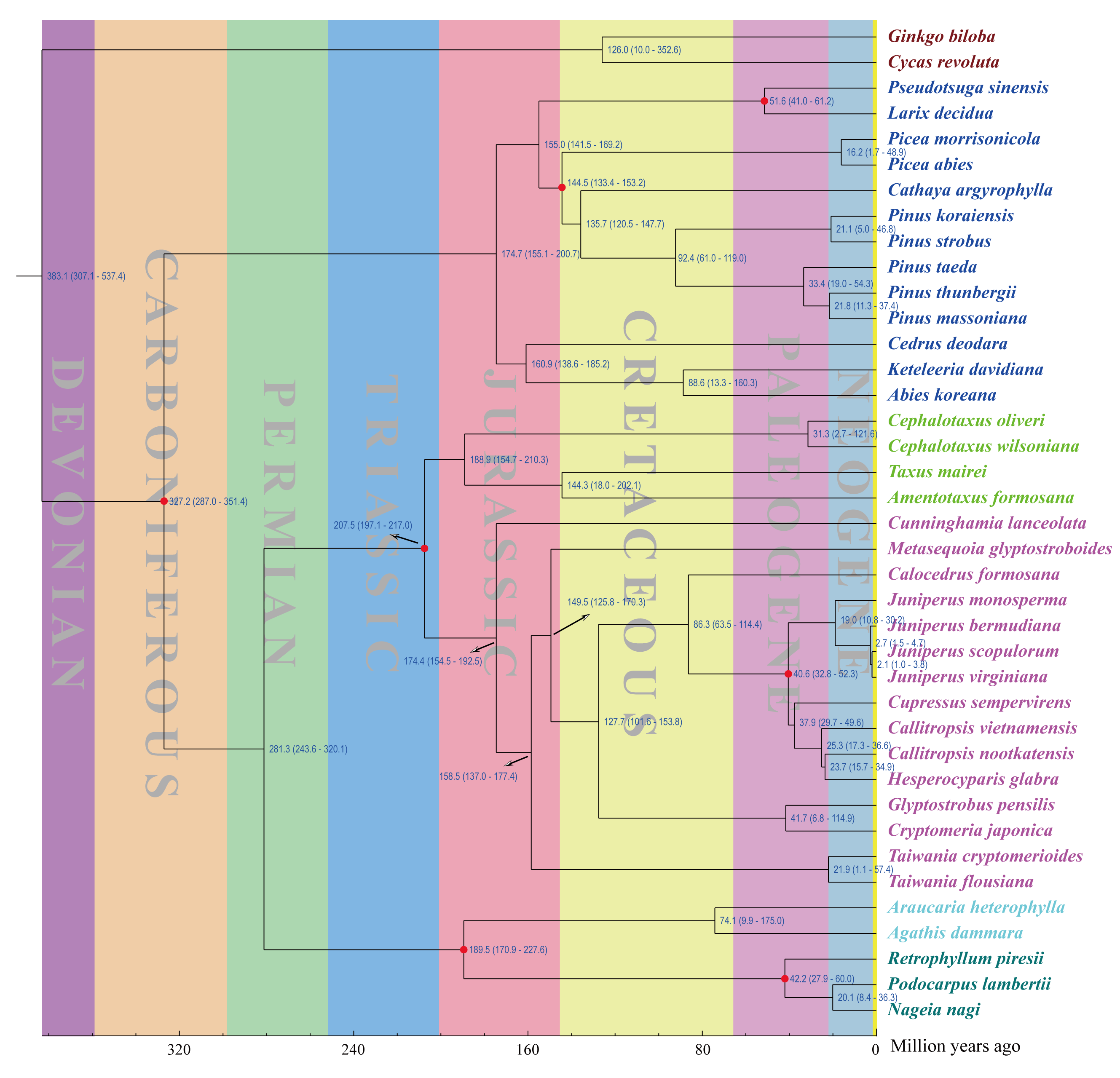

Supplement: S3 Fig — A time scale is shown at the bottom and these colored rectangles indicate different geological periods. The red points in some nodes indicate fossil calibration points. (TIF) [file pone.0161809.s004.tif]
