# Supplementary material for: The Complete Chloroplast Genome Sequence of a Relict Conifer Glyptostrobus pensilis: Comparative Analysis and Insights into Dynamics of Chloroplast Genome Rearrangement in Cupressophytes and Pinaceae
Source: PLoS One. 2016 Aug 25;11(8):e0161809. doi: 10.1371/journal.pone.0161809 (PMC4999192; doi:10.1371/journal.pone.0161809)
Supplement: S1 Table — (DOCX) [file pone.0161809.s005.docx]

**S1 Table. The validation results for the structures of regions containing three pairs of longest repeats by PCR amplicons and Sanger sequencing.**

| Primers | | Target sequence position | Palindromic repeats ^a^ | | Identities ^b^ |
| --- | --- | --- | --- | --- | --- |
| F | 5'-TCGACTGAACGCTAAAGAACCA-3' | 22060..23227 | repeat_len113 | 22771..22883 | 100% |
| R | 5'-TTGTTGCAATTGCCCAGGATAC-3' |  |  |  |  |
| F | 5'-CCCTATTGACTGCAACCTCTGT-3' | 95040..96359 |  | 96100..96212 | 100% |
| R | 5'-CTCTGACCGGACATCAAAGTGA-3' |  |  |  |  |
| F | 5'-GTGAAGGCCCCATATCCGTATT-3' | 111868..113247 | repeat_len191 | 112794..112984 | 100% |
| R | 5'-ATTAGCGAACGTTGCGAAAGAG-3' |  |  |  |  |
| F | 5'-TGGGAATTCACACGACCAAGAA-3' | 128115..129348 |  | 128595..128785 | 100% |
| R | 5'-GGCAAGTGGTTGACTTTGTACC-3' |  |  |  |  |
| F | 5'-ATTTGGCTCAGGATTCCCCATT-3' | 50010..51229 | repeat_len283 | 50236..50518 | 100% |
| R | 5'-CTGTTGATCCCTTGGCTCTTCT-3' |  |  |  |  |
| F | 5'-CCAAACTCCAAGTGTGATGCAG-3' | 87611..88625 |  | 88266..88548 | 99.47% |
| R | 5'-GGTTCCAATATGAGCAGGACCA-3' |  |  |  |  |

a. Three pairs of palindromic repeats, which were listed in Supplementary Table 8, were identified as the longest repeats in the *G. pensilis* cp genomes.

b. Identities between target sequences and PCR products sequenced by Sanger sequencing.
