# Supplementary material for: The Complete Chloroplast Genome Sequence of a Relict Conifer Glyptostrobus pensilis: Comparative Analysis and Insights into Dynamics of Chloroplast Genome Rearrangement in Cupressophytes and Pinaceae
Source: PLoS One. 2016 Aug 25;11(8):e0161809. doi: 10.1371/journal.pone.0161809 (PMC4999192; doi:10.1371/journal.pone.0161809)
Supplement: S2 Table — (DOCX) [file pone.0161809.s006.docx]

**S2 Table. GenBank accession numbers of the cp genomes used in this study.**

| **NO.** | **Taxon** | **Order** | **Family** | **Genus** | **GenBank Accession number** | **Genome Size (bp)** |
| --- | --- | --- | --- | --- | --- | --- |
| 1 | *Juniperus bermudiana* | Cupressales | Cupressaceae | Juniperus | NC_024021 | 127,659 |
| 2 | *Juniperus virginiana* | Cupressales | Cupressaceae | Juniperus | NC_024024 | 127,770 |
| 3 | *Juniperus scopulorum* | Cupressales | Cupressaceae | Juniperus | NC_024023 | 127,774 |
| 4 | *Juniperus monosperma* | Cupressales | Cupressaceae | Juniperus | NC_024022 | 127,744 |
| 5 | *Taiwania cryptomerioides* | Cupressales | Cupressaceae | Taiwania | NC_016065 | 132,588 |
| 6 | *Taiwania flousiana* | Cupressales | Cupressaceae | Taiwania | NC_021441 | 131,413 |
| 7 | *Callitropsis vietnamensis* | Cupressales | Cupressaceae | Callitropsis | NC_026298 | 127,541 |
| 8 | *Callitropsis nootkatensis* | Cupressales | Cupressaceae | Callitropsis | NC_026295 | 127,150 |
| 9 | *Calocedrus formosana* | Cupressales | Cupressaceae | Calocedrus | NC_023121 | 127,311 |
| 10 | *Cunninghamia lanceolata* | Cupressales | Cupressaceae | Cunninghamia | NC_021437 | 135,334 |
| 11 | *Cryptomeria japonica* | Cupressales | Cupressaceae | Cryptomeria | NC_010548 | 131,810 |
| 12 | *Metasequoia glyptostroboides* | Cupressales | Cupressaceae | Metasequoia | NC_027423 | 131,887 |
| 13 | *Glyptostrobus pensilis* | Cupressales | Cupressaceae | Glyptostrobus | KU_302768 | 132,239 |
| 14 | *Cupressus sempervirens* | Cupressales | Cupressaceae | Cupressus | NC_026296 | 129,150 |
| 15 | *Hesperocyparis glabra* | Cupressales | Cupressaceae | Hesperocyparis | NC_026297 | 126,993 |
| 16 | *Cephalotaxus oliveri* | Cupressales | Taxaceae | Cephalotaxus | NC_021110 | 134,337 |
| 17 | *Cephalotaxus wilsoniana* | Cupressales | Taxaceae | Cephalotaxus | NC_016063 | 136,196 |
| 18 | *Amentotaxus formosana* | Cupressales | Taxaceae | Amentotaxus | NC_024945 | 136,430 |
| 19 | *Taxus mairei* | Cupressales | Taxaceae | Taxus | NC_020321 | 127,665 |
| 20 | *Araucaria heterophylla* | Araucariales | Araucariaceae | Araucaria | NC_026450 | 146,723 |
| 21 | *Agathis dammara* | Araucariales | Araucariaceae | Agathis | NC_023119 | 145,625 |
| 22 | *Podocarpus lambertii* | Araucariales | Podocarpaceae | Podocarpus | NC_023805 | 133,734 |
| 23 | *Retrophyllum piresii* | Araucariales | Podocarpaceae | Retrophyllum | NC_024827 | 133,291 |
| 24 | *Nageia nagi* | Araucariales | Podocarpaceae | Nageia | NC_023120 | 133,722 |
| 25 | *Pinus strobus* | Pinales | Pinaceae | Pinus | NC_026302 | 115,576 |
| 26 | *Pinus thunbergii* | Pinales | Pinaceae | Pinus | NC_001631 | 119,707 |
| 27 | *Pinus taeda* | Pinales | Pinaceae | Pinus | NC_021440 | 121,530 |
| 28 | *Pinus koraiensis* | Pinales | Pinaceae | Pinus | NC_004677 | 117,190 |
| 29 | *Pinus massoniana* | Pinales | Pinaceae | Pinus | NC_021439 | 119,739 |
| 30 | *Picea abies* | Pinales | Pinaceae | Picea | NC_021456 | 124,084 |
| 31 | *Picea morrisonicola* | Pinales | Pinaceae | Picea | NC_016069 | 124,168 |
| 32 | *Cathaya argyrophylla* | Pinales | Pinaceae | Cathaya | NC_014589 | 107,122 |
| 33 | *Pseudotsuga sinensis var. wilsoniana* | Pinales | Pinaceae | Pseudotsuga | NC_016064 | 122,513 |
| 34 | *Abies koreana* | Pinales | Pinaceae | Abies | NC_026892 | 121,373 |
| 35 | *Keteleeria davidiana* | Pinales | Pinaceae | Keteleeria | NC_011930 | 117,720 |
| 36 | *Larix decidua* | Pinales | Pinaceae | Larix | NC_016058 | 122,474 |
| 37 | *Cedrus deodara* | Pinales | Pinaceae | Cedrus | NC_014575 | 119,299 |
| 38 | *Ginkgo biloba* | Ginkgoales | Ginkgoaceae | Ginkgo | NC_016986 | 156,988 |
| 39 | *Cycas revoluta* | Cycadales | Cycadaceae | Cycas | NC_020319 | 162,489 |
