# Supplementary material for: The Complete Chloroplast Genome Sequence of a Relict Conifer Glyptostrobus pensilis: Comparative Analysis and Insights into Dynamics of Chloroplast Genome Rearrangement in Cupressophytes and Pinaceae
Source: PLoS One. 2016 Aug 25;11(8):e0161809. doi: 10.1371/journal.pone.0161809 (PMC4999192; doi:10.1371/journal.pone.0161809)
Supplement: S4 Table — (DOCX) [file pone.0161809.s008.docx]

**S4 Table. The index of substitution saturation (Iss) values of 64 protein-coding genes common to 39 species.**

| **NO.** | **Gene** | **P-inva** | **Iss** | **Iss.cSym** | **Iss.cAsym** |
| --- | --- | --- | --- | --- | --- |
| 1 | *accD** | 0.1822 | 1.774 | 0.799 | 0.533 |
| 2 | *atpA* | 0.6660 | 0.339 | 0.775 | 0.493 |
| 3 | *atpB* | 0.6728 | 0.348 | 0.774 | 0.489 |
| 4 | *atpE** | 0.5441 | 0.376 | 0.692 | 0.363 |
| 5 | *atpF* | 0.5018 | 0.336 | 0.708 | 0.380 |
| 6 | *atpH* | 0.7764 | 0.325 | 0.683 | 0.359 |
| 7 | *atpI* | 0.6680 | 0.315 | 0.727 | 0.406 |
| 8 | *ccsA* | 0.5366 | 0.392 | 0.745 | 0.436 |
| 9 | *cemA* | 0.5026 | 0.335 | 0.730 | 0.410 |
| 10 | *chlB* | 0.6420 | 0.347 | 0.776 | 0.494 |
| 11 | *chlL* | 0.6808 | 0.305 | 0.738 | 0.424 |
| 12 | *chlN* | 0.6105 | 0.319 | 0.769 | 0.481 |
| 13 | *infA** | 0.2778 | 0.470 | 0.684 | 0.362 |
| 14 | *matK* | 0.4011 | 0.362 | 0.774 | 0.490 |
| 15 | *petA* | 0.6366 | 0.329 | 0.745 | 0.437 |
| 16 | *petB* | 0.7330 | 0.331 | 0.717 | 0.391 |
| 17 | *petD** | 0.7313 | 0.377 | 0.702 | 0.377 |
| 18 | *petG* | 0.7193 | 0.288 | 0.769 | 0.523 |
| 19 | *petN* | 0.7556 | 0.354 | 0.840 | 0.648 |
| 20 | *psaA* | 0.7401 | 0.316 | 0.795 | 0.527 |
| 21 | *psaB* | 0.7546 | 0.315 | 0.794 | 0.526 |
| 22 | *psaC* | 0.8008 | 0.265 | 0.683 | 0.359 |
| 23 | *psaI** | 0.3617 | 1.215 | 0.727 | 0.447 |
| 24 | *psaJ* | 0.4574 | 0.354 | 0.743 | 0.475 |
| 25 | *psbA* | 0.7825 | 0.276 | 0.752 | 0.450 |
| 26 | *psbB* | 0.7531 | 0.305 | 0.776 | 0.493 |
| 27 | *psbC* | 0.7638 | 0.308 | 0.768 | 0.480 |
| 28 | *psbD* | 0.8155 | 0.295 | 0.748 | 0.443 |
| 29 | *psbE* | 0.7381 | 0.292 | 0.683 | 0.357 |
| 30 | *psbF* | 0.7583 | 0.268 | 0.758 | 0.502 |
| 31 | *psbH* | 0.5644 | 0.313 | 0.685 | 0.364 |
| 32 | *psbI* | 0.6847 | 0.292 | 0.776 | 0.535 |
| 33 | *psbJ* | 0.5439 | 0.358 | 0.769 | 0.523 |
| 34 | *psbK* | 0.5480 | 0.395 | 0.700 | 0.395 |
| 35 | *psbL* | 0.6325 | 0.253 | 0.763 | 0.512 |
| 36 | *psbM* | 0.4381 | 0.423 | 0.791 | 0.561 |
| 37 | *psbN* | 0.7273 | 0.271 | 0.739 | 0.468 |
| 38 | *psbT* | 0.7685 | 0.387 | 0.783 | 0.548 |
| 39 | *psbZ* | 0.6984 | 0.331 | 0.695 | 0.384 |
| 40 | *rbcL* | 0.7444 | 0.304 | 0.772 | 0.486 |
| 41 | *rpl2* | 0.5706 | 0.345 | 0.734 | 0.418 |
| 42 | *rpl14* | 0.6504 | 0.320 | 0.688 | 0.358 |
| 43 | *rpl16** | 0.6473 | 0.444 | 0.693 | 0.364 |
| 44 | *rpl20** | 0.5057 | 0.355 | 0.686 | 0.356 |
| 45 | *rpl22** | 0.3440 | 0.641 | 0.692 | 0.363 |
| 46 | *rpl23** | 0.4593 | 0.480 | 0.682 | 0.355 |
| 47 | *rpl32** | 0.3594 | 0.493 | 0.744 | 0.478 |
| 48 | *rpl33* | 0.5365 | 0.324 | 0.693 | 0.382 |
| 49 | *rpl36* | 0.6140 | 0.394 | 0.769 | 0.523 |
| 50 | *rpoA* | 0.4476 | 0.386 | 0.746 | 0.439 |
| 51 | *rpoB* | 0.5330 | 0.436 | 0.806 | 0.547 |
| 52 | *rpoC1* | 0.5184 | 0.412 | 0.789 | 0.519 |
| 53 | *rpoC2** | 0.4483 | 0.903 | 0.809 | 0.556 |
| 54 | *rps2** | 0.5253 | 0.406 | 0.722 | 0.397 |
| 55 | *rps3** | 0.4882 | 0.425 | 0.716 | 0.389 |
| 56 | *rps4** | 0.4917 | 0.422 | 0.713 | 0.385 |
| 57 | *rps8* | 0.5269 | 0.339 | 0.688 | 0.359 |
| 58 | *rps11** | 0.5055 | 0.972 | 0.697 | 0.370 |
| 59 | *rps14* | 0.6033 | 0.338 | 0.683 | 0.354 |
| 60 | *rps15* | 0.4444 | 0.352 | 0.682 | 0.356 |
| 61 | *rps18** | 0.3290 | 1.591 | 0.698 | 0.371 |
| 62 | *rps19** | 0.4558 | 0.482 | 0.682 | 0.354 |
| 63 | *ycf3* | 0.7505 | 0.290 | 0.703 | 0.378 |
| 64 | *ycf4* | 0.6026 | 0.310 | 0.707 | 0.379 |

P-inva: proportion of invariant sites; Iss: an entropy-based index of substitution saturation proposed by Xia et al.; Iss.cSym: the critical Iss value assuming a symmetrical topology. Iss.cAsym: the critical Iss value assuming a asymmetrical topology. One asterisk after a gene indicate that the Iss value of this gene is greater than the Iss.cSym or Iss.cAsym value which indicated that this gene had experienced severe substitution saturation and should be excluded for the following phylogenetic analysis.
