# Supplementary material for: The Complete Chloroplast Genome Sequence of a Relict Conifer Glyptostrobus pensilis: Comparative Analysis and Insights into Dynamics of Chloroplast Genome Rearrangement in Cupressophytes and Pinaceae
Source: PLoS One. 2016 Aug 25;11(8):e0161809. doi: 10.1371/journal.pone.0161809 (PMC4999192; doi:10.1371/journal.pone.0161809)
Supplement: S5 Table — (DOCX) [file pone.0161809.s009.docx]

**S5 Table. Genes present in the *Glyptostrobus pensilis* chloroplast genome.**

|  | **Group of genes** | **Gene names** |
| --- | --- | --- |
| 1 | Photosystem I | *psaA*, *psaB*, *psaC*, *psaI*, *psaJ, psaM* |
| 2 | Photosystem II | *psbA*, *psbB*, *psbC*, *psbD*, *psbE*, *psbF*, *psbH*, *psbI*, *psbJ*, *psbK*, *psbL*, *psbM*, *psbN*, *psbT*, *psbZ* |
| 3 | Cytochrome b/f complex | *petA*, *petB**, *petD**, *petG*, *petL*, *petN* |
| 4  5 | ATP synthase  Chlorophyll biosynthesis | *atpA*, *atpB*, *atpE*, *atpF**, *atpH*, *atpI*  *chlB,chlL,chlN* |
| 5 | NADH dehydrogenase | *ndhA**, *ndhB**, *ndhC*, *ndhD*, *ndhE*, *ndhF*, *ndhG*, *ndhH*, *ndhI*, *ndhJ*, *ndhK* |
| 6 | RubisCO large subunit | *rbcL* |
| 7 | RNA polymerase | *rpoA*, *rpoB*, *rpoC1**, *rpoC2* |
| 8 | Ribosomal proteins (SSU) | *rps2*, rps3, *rps4*, *rps7*, *rps8*, *rps11*, *rps12***, *rps14*, *rps15*, *rps16**, rps18, *rps19* |
| 9 | Ribosomal proteins (LSU) | *rpl2**, *rpl14*, *rpl16**, *rpl20*, *rpl22*, *rpl23*, *rpl32*, *rpl33*, *rpl36* |
| 10 | Other genes | *accD, ccsA*, *cemA, clpP, infA, matK* |
| 11 | Proteins of unknown function | *ycf1, ycf2, ycf3***, *ycf4* |
| 12 | Transfer RNAs | 32 *tRNA*s (6 contain an intron, 2 were duplicated) |
| 13 | Ribosomal RNAs | *rrn4.5*, *rrn5*, *rrn16*, *rrn23* |

One or two asterisks after genes indicate that gene contains one or two introns, respectively.
