# Supplementary material for: The Complete Chloroplast Genome Sequence of a Relict Conifer Glyptostrobus pensilis: Comparative Analysis and Insights into Dynamics of Chloroplast Genome Rearrangement in Cupressophytes and Pinaceae
Source: PLoS One. 2016 Aug 25;11(8):e0161809. doi: 10.1371/journal.pone.0161809 (PMC4999192; doi:10.1371/journal.pone.0161809)
Supplement: S6 Table — (DOCX) [file pone.0161809.s010.docx]

**S6 Table. Genes with introns in the *G. pensilis* cp genome.**

| **Gene** | **Start** | **End** | **ExonⅠ**  **(bp)** | **IntronⅠ**  **(bp)** | **ExonⅡ**  **(bp)** | **IntronⅡ**  **(bp)** | **ExonⅢ**  **(bp)** |
| --- | --- | --- | --- | --- | --- | --- | --- |
| *atpF* | 82,139 | 83,372 | 136 | 688 | 410 |  |  |
| *ndhA* | 42,895 | 44,752 | 558 | 751 | 549 |  |  |
| *ndhB* | 25,358 | 23,196 | 723 | 771 | 756 |  |  |
| *petB* | 3,269 | 4,687 | 6 | 1,045 | 642 |  |  |
| *petD* | 4,854 | 5,999 | 8 | 648 | 490 |  |  |
| *rpl16* | 10,989 | 9,713 | 9 | 857 | 411 |  |  |
| *rpl2* | 14,195 | 12,690 | 403 | 672 | 431 |  |  |
| *rpoC1* | 72,664 | 75,462 | 442 | 702 | 1,655 |  |  |
| *rps12** | 119,057 | 26,181 | 114 | - | 232 | 531 | 26 |
| *rps16* | 90,258 | 91,264 | 40 | 884 | 83 |  |  |
| *trnA-UGC* | 31,463 | 32,307 | 38 | 772 | 35 |  |  |
| *trnG-UCC* | 86,140 | 85,227 | 24 | 791 | 49 |  |  |
| *trnI-GAU* | 31,448 | 31,411 | 42 | 887 | 35 |  |  |
| *trnK-UUU* | 91,983 | 94,461 | 30 | 2,424 | 25 |  |  |
| *trnL-UAA* | 50,139 | 49,572 | 35 | 483 | 50 |  |  |
| *trnV-UAC* | 104,006 | 103,401 | 39 | 530 | 37 |  |  |
| *ycf3* | 52,646 | 54,549 | 126 | 699 | 228 | 695 | 156 |

* *Rps12* is a trans-spliced gene
