# Supplementary material for: The Complete Chloroplast Genome Sequence of a Relict Conifer Glyptostrobus pensilis: Comparative Analysis and Insights into Dynamics of Chloroplast Genome Rearrangement in Cupressophytes and Pinaceae
Source: PLoS One. 2016 Aug 25;11(8):e0161809. doi: 10.1371/journal.pone.0161809 (PMC4999192; doi:10.1371/journal.pone.0161809)
Supplement: S7 Table — (DOCX) [file pone.0161809.s011.docx]

**S7 Table. The codon-anticodon recognition pattern and codon usage for the** ***G. pensilis* cp genome.**

| **Amino acid** | **Codon** | **No.** | **RSCU** | **tRNA** | **Amino acid** | **Codon** | **No.** | **RSCU** | **tRNA** |
| --- | --- | --- | --- | --- | --- | --- | --- | --- | --- |
| Phe | UUU | 1,003 | 1.42 |  | Tyr | UAU | 726 | 1.60 |  |
| Phe | UUC | 414 | 0.58 | *trnF-GAA* | Tyr | UAC | 180 | 0.40 | *trnY-GUA* |
| Leu | UUA | 876 | 1.98 | *trnL-UAA* | Stop | UAA | 45 | 1.63 |  |
| Leu | UUG | 542 | 1.22 | *trnL-CAA* | Stop | UAG | 19 | 0.69 |  |
| Leu | CUU | 544 | 1.23 |  | His | CAU | 408 | 1.56 |  |
| Leu | CUC | 153 | 0.35 |  | His | CAC | 114 | 0.44 | *trnH-GUG* |
| Leu | CUA | 396 | 0.89 | *trnL-UAG* | Gln | CAA | 689 | 1.57 | *trnQ-UUG* |
| Leu | CUG | 149 | 0.34 |  | Gln | CAG | 190 | 0.43 |  |
| Ile | AUU | 1,038 | 1.48 |  | Asn | AAU | 877 | 1.57 |  |
| Ile | AUC | 364 | 0.52 | *trnI-GAU* | Asn | AAC | 237 | 0.43 | *trnN-GUU* |
| Ile | AUA | 709 | 1.01 | *trnI-CAU* | Lys | AAA | 1,176 | 1.53 | *trnK-UUU* |
| Met | AUG | 586 | 1.00 | *trn(f)M-CAU* | Lys | AAG | 360 | 0.47 |  |
| Val | GUU | 448 | 1.37 |  | Asp | GAU | 862 | 1.63 |  |
| Val | GUC | 166 | 0.51 | *trnV-GAC* | Asp | GAC | 196 | 0.37 | *trnD-GUC* |
| Val | GUA | 498 | 1.52 | *trnV-UAC* | Glu | GAA | 1,108 | 1.57 | *trnE-UUC* |
| Val | GUG | 198 | 0.60 |  | Glu | GAG | 306 | 0.43 |  |
| Ser | UCU | 523 | 1.88 |  | Cys | UGU | 198 | 1.42 |  |
| Ser | UCC | 225 | 0.81 | *trnS-GGA* | Cys | UGC | 81 | 0.58 | *trnC-GCA* |
| Ser | UCA | 334 | 1.20 | *trnS-UGA* | Stop | UGA | 19 | 0.69 |  |
| Ser | UCG | 145 | 0.52 |  | Trp | UGG | 427 | 1.00 | *trnW-CCA* |
| Pro | CCU | 434 | 1.68 |  | Arg | CGU | 342 | 1.44 | *trnR-ACG* |
| Pro | CCC | 192 | 0.74 | *trnP-GGG* | Arg | CGC | 106 | 0.45 |  |
| Pro | CCA | 288 | 1.12 | *trnP-UGG* | Arg | CGA | 308 | 1.30 |  |
| Pro | CCG | 118 | 0.46 |  | Arg | CGG | 77 | 0.32 |  |
| Thr | ACU | 504 | 1.70 |  | Ser | AGU | 338 | 1.22 |  |
| Thr | ACC | 205 | 0.69 |  | Ser | AGC | 104 | 0.37 | *trnS-GCU* |
| Thr | ACA | 353 | 1.19 | *trnT-UGU* | Arg | AGA | 459 | 1.93 | *trnR-UCU* |
| Thr | ACG | 127 | 0.43 |  | Arg | AGG | 135 | 0.57 |  |
| Ala | GCU | 715 | 1.97 |  | Gly | GGU | 581 | 1.47 |  |
| Ala | GCC | 178 | 0.49 |  | Gly | GGC | 154 | 0.39 | *trnG-GCC* |
| Ala | GCA | 436 | 1.20 | *trnA-UGC* | Gly | GGA | 639 | 1.62 | *trnG-UCC* |
| Ala | GCG | 126 | 0.35 |  | Gly | GGG | 205 | 0.52 |  |

RSCU: relative synonymous codon usage.
